# Microbiological Quality Estimation of Meat Using Deep CNNs on Embedded Hardware Systems

**DOI:** 10.3390/s23094233

**Published:** 2023-04-24

**Authors:** Dimitrios Kolosov, Lemonia-Christina Fengou, Jens Michael Carstensen, Nette Schultz, George-John Nychas, Iosif Mporas

**Affiliations:** 1 School of Physics, Engineering and Computer Science, University of Hertfordshire, Hatfield AL10 9AB, UK; 2 Laboratory of Microbiology and Biotechnology of Foods, Department of Food Science and Human Nutrition, School of Food and Nutritional Sciences, Agricultural University of Athens, 11855 Athens, Greece; lefengou@aua.gr (L.-C.F.); gjn@aua.gr (G.-J.N.); 3 Videometer A/S, Hørkær 12B, 2730 Herlev, Denmark; jmc@videometer.com (J.M.C.); ns@videometer.com (N.S.)

**Keywords:** food quality, spectroscopy, multispectral imaging, embedded systems

## Abstract

Spectroscopic sensor imaging of food samples meta-processed by deep machine learning models can be used to assess the quality of the sample. This article presents an architecture for estimating microbial populations in meat samples using multispectral imaging and deep convolutional neural networks. The deep learning models operate on embedded platforms and not offline on a separate computer or a cloud server. Different storage conditions of the meat samples were used, and various deep learning models and embedded platforms were evaluated. In addition, the hardware boards were evaluated in terms of latency, throughput, efficiency and value on different data pre-processing and imaging-type setups. The experimental results showed the advantage of the XavierNX platform in terms of latency and throughput and the advantage of Nano and RP4 in terms of efficiency and value, respectively.

## 1. Introduction

Ensuring the quality and safety of food products is expected by consumers, especially for highly perishable (e.g., freshly ground meat) food commodities. Food products’ quality and safety control must be accurate and efficient to meet consumers’ increasing expectations and standards, which in turn results in more demanding and labour-intensive processes [1]. In addition, the increasing demand for food due to the increase in the world population, especially in some developing countries such as China and India [2], has made it even more challenging to ensure food quality and safety control processes.

Various food analysis methods, inspections and audits are taking place to evaluate the quality and safety of raw and processed materials and the product, considered the control measure [3,4]. In the case of monitoring microbiological food safety and quality, microbiological analysis (e.g., colony counting methods), chemical analysis, or molecular techniques are performed [5,6]. However, these methods are time-consuming, provide retrospective results, are expensive or depend on high-tech infrastructure and require specialized staff [7,8], which in turn allows microbiological inspection only of a small sample of the food products available on the market [4].

The progress over the last decades in sensors technology and Artificial Intelligence, particularly in machine learning and computer vision, has enabled the development of solutions for automatic quality and authenticity assessment of food products, like meat and fish [3,9], dairy products [10], food powders [11] or oil [12]. Such technological solutions allow the quick and low-cost assessment of the quality of food products without the need for time-consuming lab-based analysis and without the need for expert/specialised staff, which anyway can cause human errors [1].

Regarding sensors, a wide range of technologies has been used for food products’ quality and safety assessment. The most typical sensor technologies used include high-performance liquid chromatography, gas chromatography, high-performance liquid chromatography–mass spectrometry, gas chromatography–mass spectrometry (GC-MS), nuclear magnetic resonance, near-infrared (NIR), Fourier transforms infrared spectroscopy (FT-IR), and Raman spectrometry [13]. In addition, the commercial availability of portable spectroscopic-based sensors (e.g., NIR, FTIR, Raman, multi-, hyper-spectral imaging) and the current research interest towards miniaturization of the sensors [14,15] is of great importance since it could allow the real-time control of several stages of the food production on-site for quality, safety, and authentication aspects.

As regards Artificial Intelligence, the development of deep machine learning and computer vision methods over the last decades has enabled the processing of information extracted from sensors to automatically assess food products. Well-known and widely used machine learning algorithms, such as support vector machines (SVMs) [3,10,11], logistic regression [12], k-nearest neighbours [11] and convolutional neural networks (CNNs) [12,16,17,18], have been presented in the literature. However, most of the approaches for food processing using spectroscopy sensors and machine learning are operating the machine learning modelling and analysis offline on a computer using food data measurements collected in chemical and microbiological labs, apart from some ([18], Table 1 in [15]) where portable spectrometers were used. Moreover, the machine learning modelling and estimation analysis are running offline on a computer, except for [19] presenting a handheld spectroscopic device with onboard statistical data analysis, but not machine learning-based analysis. The recent development of powerful microprocessors and embedded systems has allowed the development of solutions which run the artificial intelligence algorithms on the edge device, with the most popular applications of ‘AI on edge’ being in computer vision [20,21].

This article presents an architecture for estimating microbial population on meat samples using spectroscopic images and deep machine learning models operating on board, i.e., on well-known and state-of-the-art embedded platforms. Specifically, the performance of several different hardware platforms on microbial population estimation from multispectral images of minced pork samples under different storage conditions is evaluated in terms of latency of the AI-on-the-edge models used, throughput, efficiency and value of the embedded systems used.

The remainder of this article is organised as follows. First, Section 2 presents the architecture for microbiological quality estimation of meat samples using deep learning on embedded boards. Then, in Section 3, we present the experimental setup followed, and Section 4 discusses the evaluation results. Finally, in Section 5, the conclusion of the article is provided.

## 2. System Architecture

In this section, we describe the proposed system for estimating the microbial population of food, which consists of three phases: data acquisition, offline AI training, and online operation. The modular architecture of the system allows it to be flexible. It can be applied to different types of food and expanded upon with alternative algorithmic techniques for data pre-processing on different embedded boards.

**Figure 1:**
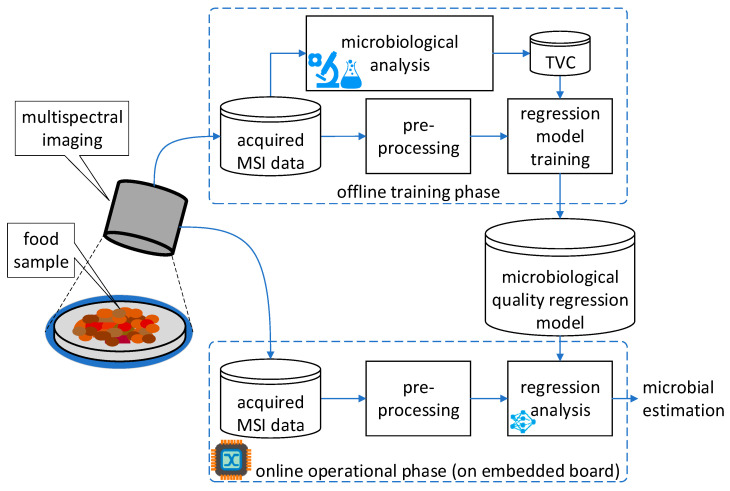
Block diagram of the food microbial population estimation architecture using multispectral imaging (MSI). The microbial population is estimated for total viable counts (TVC).

The first phase, data acquisition, involves collecting multispectral imaging (MSI) data from the food samples. The MSI data is then used in the offline AI training phase. Next, an AI model is trained to estimate the microbial population levels of the food samples using a convolutional neural network for regression (numerical estimation of the microbial population). Finally, the trained model is deployed on the edge device using an embedded hardware board in the online operation phase. It processes the MSI food images in real-time to provide microbial population estimates. Figure 1 illustrates the block diagram of the system. The following subsections provide further descriptions of each component of the architecture.

### 2.1. Acquisition of Food Imaging Data and Estimation of Microbial Population

Two datasets were formed in terms of packaging, minced pork samples that were stored aerobically (AIR) and samples that were under modified atmosphere packaging (MAP). The gas composition was 80% O_2_ and 20% CO_2._ Each dataset contained four experimental replicates (R1–R4). The samples were stored at different temperatures, from 4 °C to 12 °C and subjected to lab-based microbiological analysis. The MSI data were collected from fresh to spoiled states, covering as much as possible a representative number of samples throughout storage. The microbial population (i.e., the total viable counts, TVC) was measured using the plate count method. In parallel to the acquisition of TVC, multispectral images of the respective minced pork samples (ca. 70 g) were acquired using the VideometerLab system [22] to form a suitable dataset for the training of the machine learning regression models. Each minced meat sample was placed in a Petri dish, and the latter was placed inside an Ulbricht sphere, in which the camera was top-mounted, and the corresponding multispectral image of the product’s surface was taken. The MSI images had a resolution of 1200 × 1200 pixels and 18 different wavelengths, non-uniformly distributed ranging from 405 to 970 nm (i.e., 405, 430, 450, 470, 505, 565, 590, 630, 645, 660, 850, 870, 890, 910, 920, 940, 950 and 970 nm). A more detailed description of the data acquisition and storage conditions is available in [3].

### 2.2. Offline Training Phase

**Figure 2:**
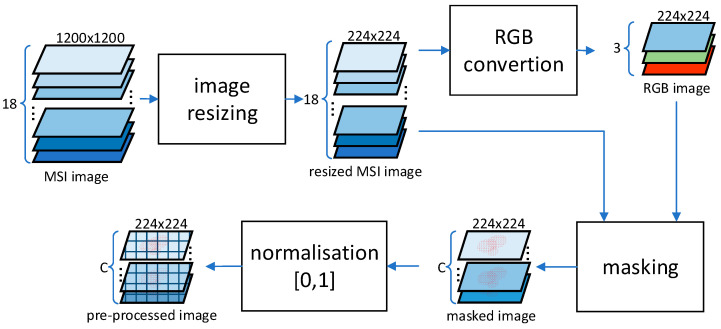
Pre-processing flow diagram of the MSI meat imaging data.

The acquired MSI meat images were pre-processed, as shown in Figure 2. The pre-processing included image resizing to 224 × 224 pixels resolution to reduce the CNN model complexity and, thus, the training time of the CNN regression models. Training on the original MSI image resolution (1200 × 1200 × 18 pixels) would result in running out of memory. Additionally, it prevents the increase of inference on edge devices, which typically have limited processing and memory resources. After resizing, the data sample is optionally converted to an RGB image (224 × 224 × 3 pixels) via concatenating specific wavelengths into a group of three channels (i.e., 645 nm for Red, 505 nm for Green, and 470 nm for Blue) to reduce further the amount of input data channels (6× times less) or remain in the MSI format (224 × 224 × 18 pixels). Next, the RGB or the MSI image is optionally masked to remove the background, petri dish and fat present in the meat images. The masking is performed by image segmentation using the k-means clustering algorithm applied to the RGB image. The resulting mask is used to segment the meat part of either the MSI or the RGB image, with the values of the pixels of the non-meat part of the images being set equal to 0 (black colour). As a final pre-processing step, pixel value normalization ([0, 1] values range) is applied, resulting in 224 × 224 × C images with C = 18 for MSI and C = 3 for RGB images.

Various machine learning regression models were evaluated to find the best-suited and most accurate deep learning architecture for predicting microbial populations. The pre-processed meat images, MSI or RGB, were used as input (X) at the training phase of the CNN regression models. At the same time, the ground truth labels (TVC values) were calculated from the microbiological analysis. During the offline training phase, various CNN models were trained for hyperparameter optimisation and evaluated using the image pre-processing steps described above.

### 2.3. Online Operation Phase

Regarding the online operation phase, the same pre-processing steps were used as in the offline phase, as shown previously in Figure 2. However, during the online operation phase, the most accurate CNN regression models were deployed on various edge devices (embedded systems) with distinct architectures. For each edge device, the regression models were optimized (quantized) for the target hardware to maximise the performance. By doing this, the model size is vastly reduced, which in turn requires fewer processing capabilities, benefiting the embedded devices’ memory requirements and compute constraints.

## 3. Experimental Setup

The experimental setup section includes a detailed description of the datasets used to train and test the CNN regression models, as well as descriptions of the models used for image segmentation and regression for microbial population estimation. It also includes a description of the edge devices benchmarked for the online operation phase and their key features.

### 3.1. Evaluation Datasets

The dataset containing multispectral images of raw minced pork was pre-processed into MSI and RGB image types, each having a set with and without masking. Each CNN model was trained on these categories using a 4-fold cross-validation experimental protocol to avoid overlapping between train and test subsets. The distribution of the AIR (424 samples) and MAP (423 samples) subsets divided into 4-fold training is tabulated in Table 1 and Table 2, respectively.

### 3.2. Models

#### 3.2.1. K-Means Masking

**Figure 3:**
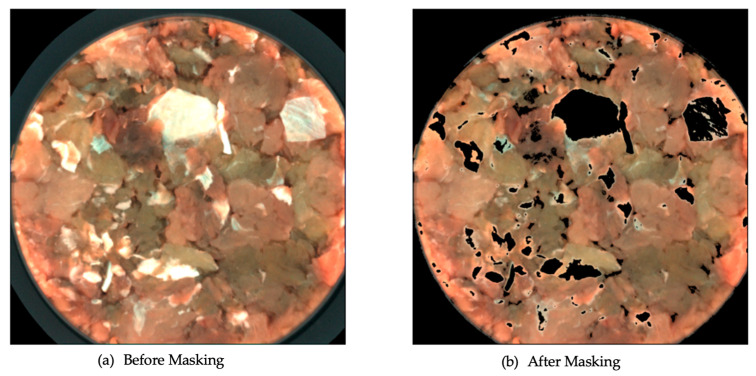
Example of the effect of k-means masking on minced pork RGB images.

To segment the minced meat images, to remove redundant information from the image samples, such as the background, the petri dish and the fat, the k-means clustering algorithm was used. By fitting a k-means model on an RGB image sample, the undesirable areas of the images were removed with an additional step of a threshold operation on the pixels. An example of the effect of masking is shown in Figure 3. The k-means model parameters were empirically optimised, and the k-means model weights were stored for reuse during the online, operational phase.

#### 3.2.2. CNN Regression Models

For the estimation of the microbiological quality of meat samples, 2D CNN regression models were used. Various well-known and widely used CNN architectures were tested, namely the MobileNet [23], DenseNet [24], EfficientNet [25], VGG16 [26], and ResNet [27]. ResNet-18 and Resnet-34 achieved significantly higher performance than the other four CNN architectures. Thus, in the remainder of this article, we considered only ResNet-based evaluations. The inputs of the ResNet-18 and ResNet-34 models were adjusted according to the input data (MSI or RGB). Finally, the output was set for regression, with detailed layer information for both architectures shown in Table 3.

### 3.3. Embedded Systems

Seven embedded systems were evaluated for this application: a 4 GB and 8 GB Raspberry Pi 4 (RP4), Intel Neural Compute Stick 2 (NCS2), and NXP i.MX 8M Plus (IMX8P), NVIDIA Jetson Nano (Nano), NVIDIA Jetson Xavier NX (XavierNX), AMD-Xilinx FPGAs Ultra96v1 (ULTRA96) and Kria (KV260). Each platform is unique regarding the underlying technology, external memory bandwidth and density, different type of AI acceleration, power consumption and cost. Table 4 presents the list of embedded systems used in the present evaluation with their core specifications, with more details on the setup explained below.

1. RP4_64bit (Raspberry Pi 4 Model B 8 GB)

The main compute element of Raspberry Pi 4 Model B is its quad-core ARM Cortex-A72 CPU that supports NEON 128-bit wide vector instructions, running at a maximum clock speed of 1.5 GHz. In addition, this variant (RP4_64bit) is fitted with 8 GB LPDDR4 and runs a 64-bit OS (Bullseye). The CNN models targeted for this embedded system were quantized (FP16, DINT8 or INT8) and inferred using TFLITE v2.8 runtime engine.

2. NCS2 (Raspberry Pi 4 Model B 4 GB + Intel Neural Compute Stick 2)

Intel NCS2 (NCS2) is a vision processing unit (VPU) accelerator with 16 low-power vector processing units 128-bit wide (a.k.a. SHAVE), running at 700 MHz. It comes in the form of a USB stick, so it does require a host controller, where an RP4 fitted with 4 GB LPDDR4 running 32bit OS (Buster) was used to act as the host. The CNN models used on NCS2 were quantized (FP16) and inferred using OpenVINO v2022.2 runtime engine.

3. IMX8P (NXP i.MX 8M Plus)

NXP i.MX 8 M Plus (IMX8P) includes a quad-core ARM Cortex-A53 running at 1.8 GHz, an ARM Cortex M7, a HiFi4 DSP running at 800 MHz, and most importantly, a Neural Processing Unit (NPU). The NPU includes several hardware features, such as a 128-bit vector engine and tensor processing cores capable of accelerating INT8 models. Any models of unsupported datatypes (e.g., FP16 and DINT8) have their inference fall back to being executed in the CPU. TFLITE v2.9.1 runtime engine was used, which meant the previous TFLITE quantized models could be reused.

4. Nano (NVIDIA Jetson Nano)

NVIDIA Jetson Nano (Nano) includes an embedded GPU with 128 CUDA cores, a quad-core ARM Cortex-A57 64-bit CPU and 4GB LPDDR4. From the two power modes supported, we used the power mode MAXN (10 Watts), where the 4× CPU cores run at 1.48 GHz and the GPU at 921.6 MHz. Running Jetpack v4.6.1, the CNN models were quantized (FP16) and executed using TensorRT (TRT) runtime engine.

5. XavierNX (NVIDIA Jetson Xavier NX)

NVIDIA Jetson Xavier NX (XavierNX) is a more powerful family than Nano, as it includes more GPU cores, a more powerful CPU, and higher density and speed LPDDR4. Its GPU comprises 384 cores and 48 Tensor Cores, while its CPU is a 64-bit 6-core NVIDIA Carmel ARMv8.2. From the various power modes, we used power mode 1 (15 watts, 4 cores), where the 4× CPUs were running 1.4 GHz and the GPU at 1.1 GHz. Running Jetpack v5.0.2, CNN models were quantized (FP16/INT8) and executed using TensorRT (TRT) runtime engine.

6. Ultra96 (Avnet Ultra96-V1)

Avnet Ultra96-V1 (Ultra96) is an AMD-Xilinx FPGA fitted with a ZU3EG variant, capable of accelerating AI models using a soft Deep Learning Processor Unit (DPU) in the Programmable Logic (PL). The DPU architecture is configurable with various parallelism and performance settings at the expense of PL resources. The Ultra96 was configured with the B1600 variant of DPUCZDX8G running at 300 MHz. The models were quantized (INT8) using Vitis-AI v2.5 and inferred with VART runtime engine.

7. KV260 (Xilinx Kria KV260 Starter Kit)

Xilinx Kria KV260 is System-on-Module with a carrier card containing an FPGA with a higher resource count than Ultra96, aimed for vision AI application. Similarly, to the Ultra96 setup, a DPU was implemented, but the main difference was configured with a more capable B4096 variant running at 300 MHz.

## 4. Experimental Results

The architecture presented in Section 2 was evaluated according to the experimental setup presented in Section 3. In Section 4.1, the performance metrics used in the training phase are outlined, the microbial population estimation results using three different CNN models on the minced pork dataset are presented, and the quantization loss results for the target edge devices are explored. Finally, in Section 4.2, the metrics used to evaluate the online (edge device) microbial population estimation and the results obtained from benchmarking each edge device on the proposed architecture, as illustrated in Figure 1.

### 4.1. Microbial Population Estimation

#### 4.1.1. Accuracy Metrics

The metrics used to evaluate the performance of the CNN regression models are the Root Mean Square Error (RMSE), the Pearson Correlation Coefficient (r), the Mean Absolute Error (MAE), and the Residual Prediction Deviation (RPD), which have also been used as the performance metrics in [3,35,36,37,38]. The equations of the metrics are described below:

(1) $$MAE=\frac{1}{N}\sum_{n=1}^{N}\left|\tilde{y_{n}}-y_{n}\right|$$

(2) $$RMSE=\frac{1}{N}\sum_{n=1}^{N}{\left(\tilde{y_{n}}-y_{n}\right)}^{2}$$

(3) $$r=\frac{\sum_{n=1}^{N}\left(y_{n}-\bar{y})(\tilde{y_{n}}-\bar{\tilde{y}}\right)}{\sqrt{\sum_{n=1}^{N}{\left(y_{n}-\bar{y}\right)}^{2}{\left(\tilde{y_{n}}-\bar{y}\right)}^{2}}}$$

(4) $$RPD=\frac{\sigma_{\tilde{y}}}{RMSE}=\frac{1}{RMSE}\sqrt{\frac{1}{N}\sum_{n=1}^{N}{\left(\tilde{y_{n}}-\bar{\tilde{y}}\right)}^{2}}$$

where $y_{n}$ is the real TVC value of the n-th meat sample as calculated from the microbiological analysis, $\tilde{y_{n}}$ is the TVC value estimated by the CNN regression model, $\bar{y}$ is the average real TVC value, $\bar{\tilde{y}}$ is the average estimated TVC value, and $\sigma_{\tilde{y}}$ is the standard deviation of the estimated TVC values.

#### 4.1.2. CNN-Based Microbial Population Estimation

The training was performed using k-fold cross-validation, with k = 4, due to the number of available replicates for each AIR and MAP dataset. The type of data that the regression models were trained with were MSI (224 × 224 × 18) or RGB (224 × 224 × 3) images, with two different pre-processing types, i.e., with masking and without masking. The training was implemented with the Root Mean Squared Propagation (RMSprop) optimizer and Mean Squared Error (MSE) as the loss function.

Table 5 and Table 6 present the averaged 4-fold cross-validation results for the AIR and MAP data. The results indicate that most CNN models have comparable performance regarding r, RMSE, and MAE metrics. However, ResNet-18 on MSI data with masking achieved the highest RPD metric. Notably, better results were observed in the AIR data than the MAP, with masking improving the RPD results on average by approximately 2.5% for AIR and 4.0% for MAP data. Some reasons for the better performance of the model based on AIR data compared to MAP data may be the different batches (e.g., initial microbial population) and the dominance of the different microbial groups due to different packaging conditions. It needs to be stressed that there is an assembly of quality characteristics of the samples contributing to the development of models. For example, as expected, the obtained better performance of the models based on aerobic samples could not be attributed to the colour since this was maintained better in the samples stored under MAP due to high oxygen presence.

Although ResNet-34 has twice as many hyperparameters as ResNet-18, slight overfitting was observed, indicating that the model may have been too complex for the given task. Furthermore, for the RGB data, despite the input data being six times less than MSI (3 channels instead of 18), the RMSE, MAE, and r values were near the MSI-based CNN model results. However, the RPD was much lower for the RGB image data.

The region from 405 nm (VIS) to 970 nm (NIR) is associated with protein, fat, and moisture [39]. Therefore, it is more informative for the ‘description’ of meat deterioration compared to changes only of colour (RGB models). Hyperspectral imaging (HSI) and multispectral imaging have been used for the prediction of freshness, quality, and safety parameters, with the region 400–1000 nm being the most utilized in animal-origin foods [40,41]. In the case of HSI, feature selection methods are applied to select key wavelengths to improve performance and computational time, and avoid overfitting [42,43]. The results of these studies show the potential in a wide range of applications. The studies by [44,45] showed great potential for the prediction of quality (i.e., TVC and TVB-N) in terms of RPD (>3). In the present study, the highest RPD was 2.83. Comparing the data acquisition workflow, in the present study, the samples were stored simulating real-life life conditions in a range of storage temperatures from 4 °C (refrigeration conditions) to 12 °C (abusive temperature), including a high number of samples (n > 400 for each packaging condition) and independent batches (R1–R4).

#### 4.1.3. CNN Performance with Data Quantization

After evaluating the FP32 data type CNN regression models, the next step was to quantize each model for the target embedded system and its compatible run time engine. As mentioned previously in Section 3.3, each hardware supports specific data types. The results of the quantization were averaged across all CNN models and compared to the original results (FP32 data), with Table 7 showing the delta change in each metric. For the metrics r and RPD, the higher is, the better, while the opposite applies to RMSE and MAE. While for the case of FP16, no loss was observed, PTQ-INT8 (Post Training Quantization) models did show a slight drop in accuracy except TRT (Xavier NX), which showed a huge drop and was unsuitable for further hardware testing. As for the rest of the INT8 results (orange coloured), the delta in loss could be minimized further via Quantization Aware Training (QAT). However, this was not explored further as the results were satisfactory to proceed with hardware evaluations.

### 4.2. Embedded Systems Performance

#### 4.2.1. Hardware Performance Metrics

Following the evaluation and comparison of the performance of each embedded system, the following main metrics were defined and used:

1. Latency: Execution time from start to finish of a specific stage. To accurately extract this measurement, the application was run multiple times, and the average latency time was calculated for each stage. The overall test time was at least 30 s; apart from the target application process, other OS processes use the hardware resources too (such as CPU cores, cache memory, etc.), which may add noise to the experimental results. Stages of interest included loading MSI data, pre-processing, and model inference.
2. Throughput

Calculating the maximum sample per second throughput of each embedded system, considering all stages of the data pipeline, but not including the loading of models.

(5) $$Throughput=\frac{1}{\left(Read\;MSI\right)+\left(Pre-Process\right)+(Model\;Inference)}$$

3. Efficiency (Throughput/Watt)

Measuring the throughput per watt each embedded system can offer.

(6) $$Efficiency=\frac{Throughput}{Runtime\;Power\;Consumption}$$

4. Value (Throughput/Cost)

Measuring the through per cost ($) each embedded system can offer.

(7) $$Value=\frac{Throughput}{Hardware\;Cost\;in\;USD}$$

#### 4.2.2. Hardware Evaluation Results

The hardware performance of each embedded system used in the study was evaluated using the metrics described above: latency, throughput, efficiency, and value. Latency is measured for each stage of the data pipeline, while throughput is calculated by considering the entire pipeline.

1. Load CNN Model (Latency)

**Figure 4:**
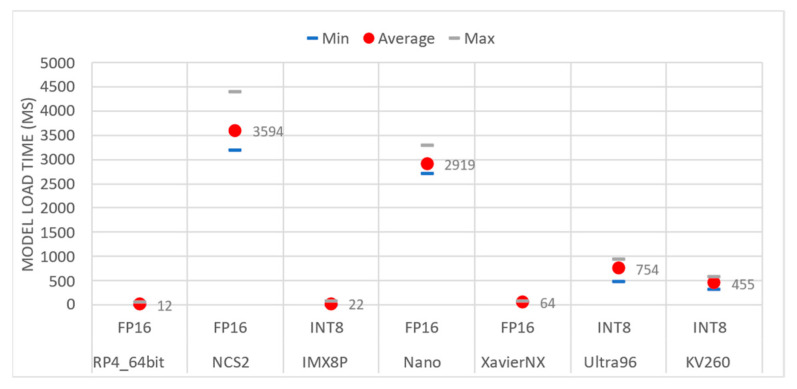
Time is required by the embedded boards to load the CNN models.

Loading the CNN model is performed once, and Figure 4 demonstrates that it could be time-consuming. The NCS2 and Nano platforms were the slowest, requiring an average of 3.6 and 2.9 s, respectively, to load the CNN model weights. In contrast, the remaining platforms required between 12 ms (RP4_64bit) and 754 ms (Ultra96). This variation in loading time depends on both the CPU capabilities and the size and data type of the quantized model.

2. Load k-means Model (Latency)

**Figure 5:**
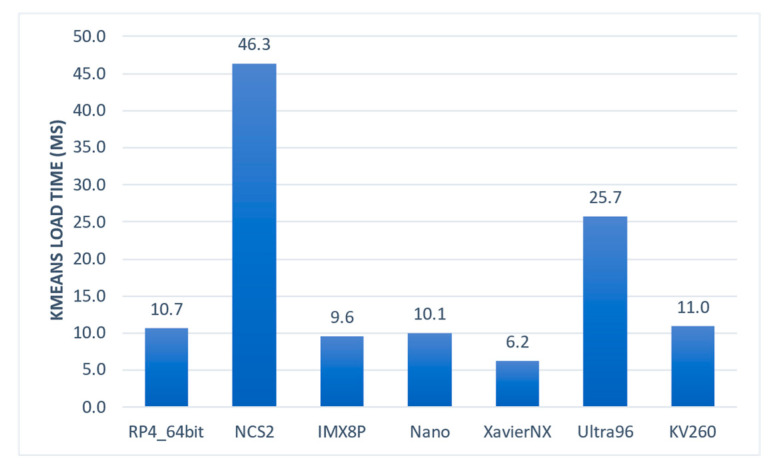
Time required by the embedded systems to load the k-means model.

When masking was used, the k-means model needed to be loaded once. After that, the model weights were binarized for re-usage, and in this stage, they were deserialized. The deserialization process depended solely on the CPU and the corresponding operating system. The NCS2 platform, which uses RP4 with a 32-bit OS, had the slowest deserialization time by a significant margin (46.3 ms). In contrast, the XavierNX, which has the latest and fastest ARM CPU with a 64-bit OS, had the fastest deserialization time (6.2 ms). Figure 5 shows the results.

3. Read MSI Samples (Latency)

**Figure 6:**
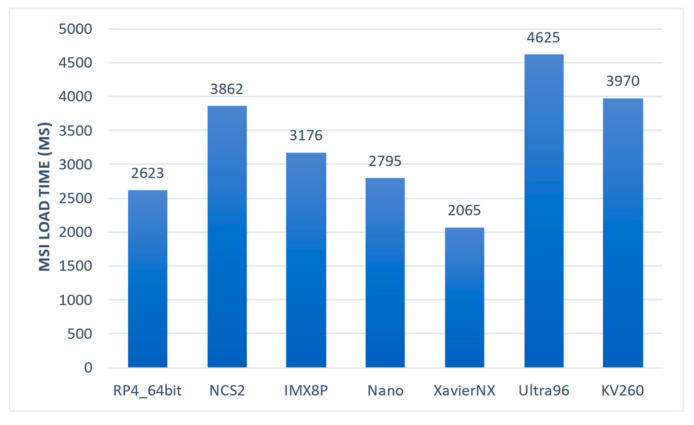
Time required by the embedded systems to load an MSI sample.

Loading a multi-spectral image was found to be the most computationally intensive task of the data pipeline, posing a significant bottleneck. The size of the MSI sample, which typically had a resolution of 1200 × 1200 × 18 pixels and an average sample size of 100 MB, further intensified the computational load. The results of loading the MSI samples across different embedded systems are presented in Figure 6. It was observed that the slowest system was the Ultra96, with an average loading time of 4.6 s, while XavierNX was the fastest one, taking only 2 s. The loading time depended on the CPU’s capabilities, particularly the clock frequency.

4. Pre-Processing (Latency)

**Figure 7:**
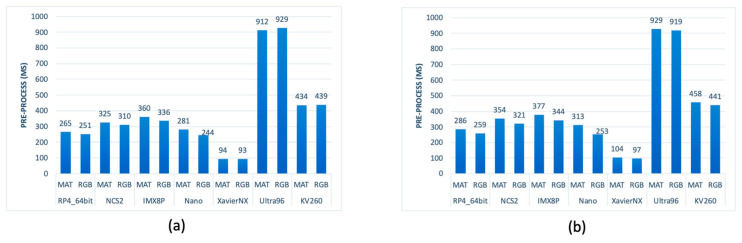
Time required by the embedded systems to pre-process the MSI and RBG images (**a**) without masking and (**b**) with masking.

The pre-processing stage of the data pipeline involved several steps (refer to Figure 2) and varied depending on the input data type, whether it was MSI or RGB image, and whether it was with or without masking. Masking had a negligible impact on pre-processing time, as shown in Figure 7, due to the resizing of the input data at the start of the pipeline, which reduced the amount of data to be processed in subsequent stages. Processing RGB data, consisting of three channels, was slightly faster than the MSI data, which had 18 channels, although the difference was not proportional. Among the platforms tested, the fastest was the XavierNX, which had the most powerful CPU, while the slowest was the Ultra96, which had the least powerful CPU.

5. CNN Regression Model Inference Time (Latency)

The results of the CNN model inference time for each embedded system, using the fastest-performing quantization with minimal quantization loss, are presented in Table 8. The ResNet models were derived from the same architecture but with varying numbers of parameters due to input shapes. The model with the least parameters (ResNet-18: RGB) was the fastest. In addition, platforms that used TFLITE models (RP4_64bit and IMX8P) could perform thread execution, further decreasing latency. The slowest platform was the IMX8P (174.8/281.7/129.3 ms), while the fastest was the XavierNX (4.3/5.9/3.0 ms) for ResNet-18:MSI, ResNet-34:MSI and ResNet18:RGB respectively.

6. Throughput (samples per second)

**Figure 8:**
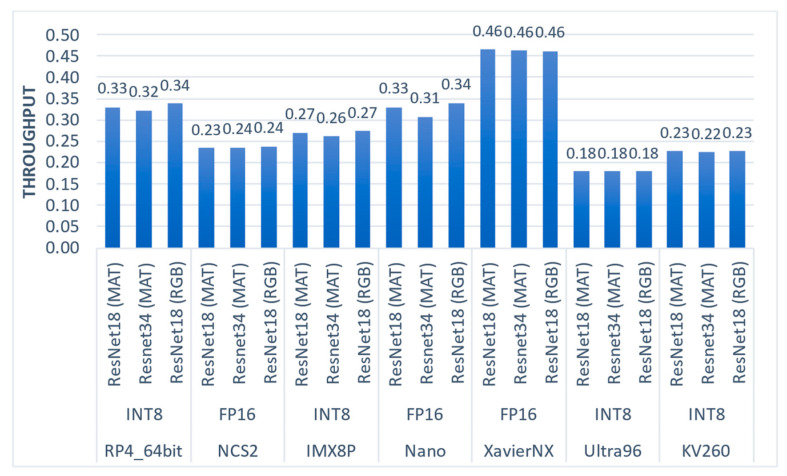
Throughput results of the evaluated embedded systems and CNN regression models.

We also evaluated the performance of the full data pipeline, considering all stages, including loading the meat sample image, pre-processing, and model inference. The total throughput results, measured in samples per second, are presented in Figure 8. Our results indicate that the loading of the meat sample image was the biggest bottleneck, negatively impacting the overall performance. Notably, the impact of masking on performance was smaller, as it affected the pre-processing stage, which was the second biggest bottleneck. The slowest platform was the Ultra96, which had the slowest CPU, while the fastest platform was the XavierNX, which had the fastest CPU. Overall, our findings suggest that the performance of this application is highly dependent on CPU capabilities.

7. Efficiency (throughput per watt)

**Figure 9:**
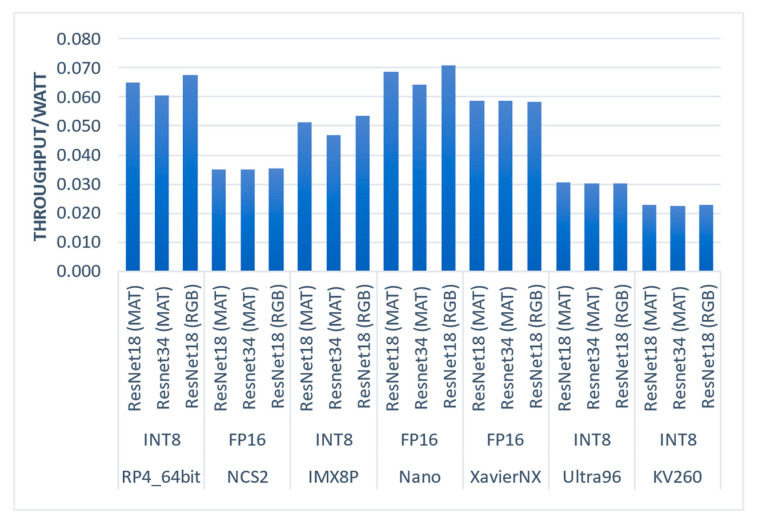
Efficiency (Throughput/Watt) of the evaluated embedded systems and CNN regression models.

Figure 9 presents each embedded system’s efficiency (samples/watts) results when considering power consumption. Based on our results, the most efficient platform was the Nano, closely followed by the RP4_64bit. On the other hand, the least efficient platforms were the KV260 and Ultra96. Interestingly, XavierNX, which performed best in latency due to its fast CPU and accelerator, ranked third in efficiency. It is worth noting that further optimization can be achieved through hardware and software/firmware optimizations, as development kits often include features that may not be necessary for a given application.

8. Value (throughput per dollar)

**Figure 10:**
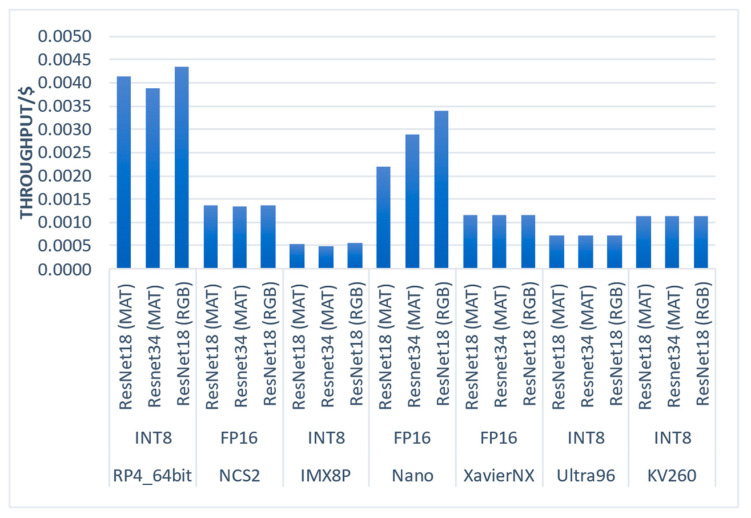
Value (Throughput/$) of the evaluated embedded systems and CNN regression models.

Finally, we measured the value metric of each platform, which considers the cost of the embedded systems (development kit). The results are presented in Figure 10, where the RP4_64bit was the most cost-efficient platform with the highest throughput per dollar. In contrast, the IMX8P was the least cost-efficient platform on the evaluated metric. Interestingly, XavierNX ranked first in the latency metrics and was observed fifth in value, indicating that it may not be the best choice for cost-sensitive applications.

## 5. Conclusions

An architecture for estimating the microbial population of food samples using multispectral imaging and deep machine learning models for regression operating on embedded hardware was presented. Minced pork samples from different storage conditions were trained using different image pre-processing techniques on AIR and MAP storage conditions and deployed on a wide range of well-known hardware platforms. The evaluation showed that the most accurate results were achieved in AIR and MAP storage conditions when applying transfer learning on the ResNet-18 model with the masked MSI images. In addition, processing RGB images instead of the MSI ones resulted in lower latency and higher throughput of the tested embedded boards, with a slight reduction of the microbial population estimation accuracy. Regarding hardware performance, the XavierNX platform outperformed all other evaluated embedded boards regarding latency and throughput because of its advantageous CPU and accelerator. In terms of energy efficiency and value, Nano and RP4 outperformed the other tested hardware boards. Moreover, on average, the loading of the MSI data corresponded to 86% of the total execution time in the end-to-end pipeline, 8% of the execution time was for the pre-processing and 6% for the inferencing of the CNN models.

The evaluation results indicate the potential of portable devices for food quality assessment using spectroscopic sensors and AI on edge. Such portable devices will allow easy and rapid testing of food quality from the corresponding public authorities in the short term and, with the further development of spectroscopic sensor technologies, also from individual consumers in the longer term.

## Figures and Tables

**Table 1:** Train and test data sets used for model development, collected during AIR storage. R stands for the replicate subset, and TVC for the total viable count (i.e., microbial population).

Train Set (Number of Samples)	Train TVC Range log CFU/g	Test Set (Number of Samples)	Test TVC Range log CFU/g
R1, R2, R3 (312)	3.08–10.32	R4 (112)	3.08–9.45
R1, R2, R4 (312)	2.00–9.93	R3 (112)	3.60–9.90
R1, R3, R4 (324)	2.00–10.32	R2 (100)	3.36–9.93
R2, R3, R4 (324)	2.00–10.32	R1 (100)	3.08–9.80

**Table 2:** Train and test data sets used for model development, collected during MAP storage. R stands for the replicate subset, and TVC for the total viable count (i.e., microbial population).

Train Set (Number of Samples)	Train TVC Range log CFU/g	Test Set (Number of Samples)	Test TVC Range log CFU/g
R1, R2, R3 (308)	3.76–9.45	R4 (115)	3.81–9.37
R1, R2, R4 (307)	2.30–9.37	R3 (116)	3.76–9.22
R1, R3, R4 (323)	2.30–9.45	R2 (100)	4.60–8.90
R2, R3, R4 (331)	2.30–9.45	R1 (92)	4.70–8.70

**Table 3:** ResNet-18 and ResNet-34 CNN model architectures.

Layer Name	Output Size	ResNet-18	ResNet-34
input	$224\times 224\times C^{\;*}$	-	
conv_1x	112×112	7×7,64,stride 2	
conv_2x	56×56	3×3,max⁡pool,stride 2	
		$\left[\begin{array}{c} 3\times 3,64\\ 3\times 3,64 \end{array}\right]\times 2$	$\left[\begin{array}{c} 3x\times 3,64\\ 3\times 3,64 \end{array}\right]\times 3$
conv_3x	28×28	$\left[\begin{array}{c} 3\times 3,128\\ 3\times 3,128 \end{array}\right]\times 2$	$\left[\begin{array}{c} 3\times 3,128\\ 3\times 3,128 \end{array}\right]\times 4$
conv_4x	14×14	$\left[\begin{array}{c} 3\times 3,256\\ 3\times 3,256 \end{array}\right]\times 2$	$\left[\begin{array}{c} 3\times 3,256\\ 3\times 3,256 \end{array}\right]\times 6$
conv_5x	7×7	$\left[\begin{array}{c} 3\times 3,512\\ 3\times 3,512 \end{array}\right]\times 2$	$\left[\begin{array}{c} 3\times 3,512\\ 3\times 3,512 \end{array}\right]\times 3$
output	1×1	average pool, linear	

* C is number of channels; C = 3 for RGB images; C = 18 for MSI images.

**Table 4:** List of embedded systems and their core specifications.

#	Hardware	CPU	Memory	AI Accelerator	AI Runtime Engine
1	RP4_64bit [28]	ARM Cortex-A72	8 GB (LPDDR4)	N/A *	TFLITE
2	RP4_32bit [28]	ARM Cortex-A72	4 GB (LPDDR4)	N/A *	OpenVINO
	NCS2 [29]	N/A	500 MB (Internal)	VPU	
3	IMX8P [30]	ARM Cortex-A53	4 GB (LPDDR4)	NPU	TFLITE
4	Nano [31]	ARM Cortex-A57	4 GB (LPDDR4)	GPU: 128-core Maxwell	TensorRT
5	XavierNX [32]	Carmel ARM^®^v8.2	8 GB (LPDDR4)	GPU: 384-core Volta	TensorRT
6	Ultra96 [33]	ARM Cortex-A53	2 GB (LPDDR4)	PL: DPU (B1600)	VART
7	KV260 [34]	ARM Cortex-A53	4 GB (LPDDR4)	PL: DPU (B4096)	VART

* N/A = Not Applicable.

**Table 5:** CNN Model Performance of microbial population estimation under AIR storage conditions; averaged results from 4-fold cross-validation on the test sets.

Model	Parameters	Type	Masking	r	RMSE	MAE	RPD
ResNet-18	11,186,625	RGB	NO	0.86	0.07	0.08	1.71
ResNet-18	11,233,665	MSI	NO	0.95	0.07	0.05	2.83
ResNet-34	21,349,249	MSI	NO	0.95	0.07	0.05	2.72
ResNet-18	11,186,625	RGB	YES	0.86	0.11	0.08	1.76
ResNet-18	11,233,665	MSI	YES	0.94	0.07	0.05	2.90
ResNet-34	21,349,249	MSI	YES	0.95	0.09	0.05	2.68

r: Pearson correlation coefficient; RMSE: root means squared error; MAE: mean absolute error; RPD: residual prediction deviation.

**Table 6:** CNN Model Performance of microbial population estimation under MAP storage conditions; averaged results from 4-fold cross-validation on the test sets.

Model	Parameters	Type	Masking	r	RMSE	MAE	RPD
ResNet-18	11,186,625	RGB	NO	0.80	0.09	0.07	1.37
ResNet-18	11,233,665	MSI	NO	0.90	0.06	0.05	1.81
ResNet-34	21,349,249	MSI	NO	0.89	0.07	0.05	1.84
ResNet-18	11,186,625	RGB	YES	0.76	0.08	0.07	1.29
ResNet-18	11,233,665	MSI	YES	0.89	0.09	0.05	1.88
ResNet-34	21,349,249	MSI	YES	0.89	0.11	0.06	1.68

r: Pearson correlation coefficient; RMSE: root mean squared error; MAE: mean absolute error; RPD: residual prediction deviation.

**Table 7:** Averaged performance Δ change for all quantizations versus original FP32 results.

Type	Data Type	r	RMSE	MAE	RPD
TF	FP32 (original)	0.89	0.08	0.06	2.04
TFLITE	FP16	0.00	0.00	0.00	0.00
TFLITE	DINT8	0.00	0.00	0.00	0.01
TFLITE	PTQ-INT8 *	0.03	−0.03	−0.03	0.35
OpenVINO	FP16	0.00	0.00	0.00	0.04
TRT	FP16	0.00	0.00	0.00	0.00
TRT	PTQ-INT8	0.27	−0.07	−0.06	1.33
VITIS	PTQ-INT8	0.02	−0.02	−0.02	0.30
VART	PTQ-INT8	0.01	−0.02	−0.02	0.25

* PTQ-INT8: Post Training Quantization. Green coloured results is for minimal delta change. Orange coloured results is for slight delta change. Red coloured results is for large delta change and not suitable.

**Table 8:** CNN model inference time for different embedded systems.

Platform	Data Type	Model	Type	Inference Time (ms)			
				1×	2×	3×	4×
RP4_64bit *	INT8	ResNet-18	MSI	316.6	189.0	144.0	125.9
		ResNet-34	MSI	530.3	312.3	231.9	200.0
		ResNet-18	RGB	238.2	140.7	105.6	92.8
NCS2	FP16	ResNet-18	MSI	61.7	-	-	-
		ResNet-34	MSI	79.2	-	-	-
		ResNet-18	RGB	24.5	-	-	-
IMX8P *	INT8	ResNet-18	MSI	534.7	293.7	212.8	174.8
		ResNet-34	MSI	913.5	493.6	352.7	281.7
		ResNet-18	RGB	404.9	221.9	160.2	129.3
Nano	FP16	ResNet-18	MSI	18.3	-	-	-
		ResNet-34	MSI	26.9	-	-	-
		ResNet-18	RGB	12.1	-	-	-
XavierNX	FP16	ResNet-18	MSI	4.3	-	-	-
		ResNet-34	MSI	5.9	-	-	-
		ResNet-18	RGB	3.0	-	-	-
Ultra96	INT8	ResNet-18	MSI	39.0	-	-	-
		ResNet-34	MSI	52.7	-	-	-
		ResNet-18	RGB	20.6	-	-	-
KV260	INT8	ResNet-18	MSI	19.7	-	-	-
		ResNet-34	MSI	22.8	-	-	-
		ResNet-18	RGB	6.9	-	-	-

* Platforms RP4_64bit and IMX8P can run multiple threads (up to maximum CPU count) with TFLITE runtime engine.

## Data Availability

The data presented in this study are available upon request. The data are not publicly available due to privacy restrictions.

## References

[1] Zhu L., Spachos P., Pensini E., Plataniotis K.N. (2021). Deep learning and machine vision for food processing: A survey. Curr. Res. Food Sci..

[2] Bodirsky B.L., Rolinski S., Biewald A., Weindl I., Popp A., Lotze-Campen H. (2015). Global Food Demand Scenarios for the 21st Century. PLoS ONE.

[3] Fengou L.-C., Mporas I., Spyrelli E., Lianou A., Nychas G.-J. (2020). Estimation of the Microbiological Quality of Meat Using Rapid and Non-Invasive Spectroscopic Sensors. IEEE Access.

[4] Nychas G.-J.E., Panagou E.Z., Mohareb F. (2016). Novel approaches for food safety management and communication. Curr. Opin. Food Sci..

[5] Bhunia A.K. (2014). One day to one hour: How quickly can foodborne pathogens be detected?. Future Microbiol..

[6] Doulgeraki A.I., Nychas G.-J.E. (2013). Monitoring the succession of the biota grown on a selective medium for pseudomonads during storage of minced beef with molecular-based methods. Food Microbiol..

[7] Munir M.T., Yu W., Young B.R., Wilson D.I. (2015). The current status of process analytical technologies in the dairy industry. Trends Food Sci. Technol..

[8] van den Berg F., Lyndgaard C.B., Sørensen K.M., Engelsen S.B. (2013). Process Analytical Technology in the food industry. Trends Food Sci. Technol..

[9] Govari M., Tryfinopoulou P., Parlapani F.F., Boziaris I.S., Panagou E.Z., Nychas G.-J.E. (2021). Quest of Intelligent Research Tools for Rapid Evaluation of Fish Quality: FTIR Spectroscopy and Multispectral Imaging Versus Microbiological Analysis. Foods.

[10] El Orche A., Mamad A., Elhamdaoui O., Cheikh A., El Karbane M., Bouatia M. (2021). Comparison of Machine Learning Classification Methods for Determining the Geographical Origin of Raw Milk Using Vibrational Spectroscopy. J. Spectrosc..

[11] Ozturk S., Bowler A., Rady A., Watson N.J. (2023). Near-infrared spectroscopy and machine learning for classification of food powders during a continuous process. J. Food Eng..

[12] Zhao H., Zhan Y., Xu Z., Nduwamungu J.J., Zhou Y., Powers R., Xu C. (2022). The application of machine-learning and Raman spectroscopy for the rapid detection of edible oils type and adulteration. Food Chem..

[13] Nychas G.-J., Sims E., Tsakanikas P., Mohareb F. (2021). Data Science in the Food Industry. Annu. Rev. Biomed. Data Sci..

[14] Rodriguez-Saona L., Aykas D.P., Borba K.R., Urtubia A. (2020). Miniaturization of optical sensors and their potential for high-throughput screening of foods. Curr. Opin. Food Sci..

[15] McVey C., Elliott C.T., Cannavan A., Kelly S.D., Petchkongkaew A., Haughey S.A. (2021). Portable spectroscopy for high throughput food authenticity screening: Advancements in technology and integration into digital traceability systems. Trends Food Sci. Technol..

[16] Chen X., Cheng G., Liu S., Meng S., Jiao Y., Zhang W., Liang J., Zhang W., Wang B., Xu X. (2022). Probing 1D convolutional neural network adapted to near-infrared spectroscopy for efficient classification of mixed fish. Spectrochim. Acta Part A Mol. Biomol. Spectrosc..

[17] Pu H., Yu J., Sun D.-W., Wei Q., Shen X., Wang Z. (2023). Distinguishing fresh and frozen-thawed beef using hyperspectral imaging technology combined with convolutional neural networks. Microchem. J..

[18] Moon E.J., Kim Y., Xu Y., Na Y., Giaccia A.J., Lee J.H. (2020). Evaluation of Salmon, Tuna, and Beef Freshness Using a Portable Spectrometer. Sensors.

[19] Karunathilaka S.R., Yakes B.J., He K., Brückner L., Mossoba M.M. (2018). First use of handheld Raman spectroscopic devices and on-board chemometric analysis for the detection of milk powder adulteration. Food Control..

[20] Kolosov D., Mporas I. Face Masks Usage Monitoring for Public Health Security using Computer Vision on Hardware. Proceedings of the 2021 International Carnahan Conference on Security Technology (ICCST).

[21] Kolosov D., Kelefouras V., Kourtessis P., Mporas I. (2022). Anatomy of Deep Learning Image Classification and Object Detection on Commercial Edge Devices: A Case Study on Face Mask Detection. IEEE Access.

[22] Carstensen J.M., Folm-Hansen J. (1999). An Apparatus and a Method of Recording an Image of an Object. Google Patents.

[23] Sandler M., Howard A., Zhu M., Zhmoginov A., Chen L.-C. MobileNetV2: Inverted Residuals and Linear Bottlenecks. Proceedings of the 2018 IEEE/CVF Conference on Computer Vision and Pattern Recognition.

[24] Huang G., Liu Z., Van Der Maaten L., Weinberger K.Q. Densely Connected Convolutional Networks. Proceedings of the 2017 IEEE Conference on Computer Vision and Pattern Recognition (CVPR).

[25] Tan M., Le Q.V. EfficientNet: Rethinking Model Scaling for Convolutional Neural Networks. Proceedings of the International conference on Machine Learning (PMLR).

[26] Simonyan K., Zisserman A. Very Deep Convolutional Networks for Large-Scale Image Recognition. Proceedings of the International Conference on Learning Representations (ICLR).

[27] He K., Zhang X., Ren S., Sun J. Deep Residual Learning for Image Recognition. Proceedings of the 2016 IEEE Conference on Computer Vision and Pattern Recognition (CVPR).

[28] Raspberry Pi 4 Model B Specifications. https://www.raspberrypi.org/products/raspberry-pi-4-model-b/.

[29] Intel Neural Compute Stick 2 Product Specifications. https://ark.intel.com/content/www/us/en/ark/products/140109/intel-neural-compute-stick-2.html.

[30] Evaluation Kit for the i.MX 8M Plus Applications Processor. https://www.nxp.com/design/development-boards/i-mx-evaluation-and-development-boards/evaluation-kit-for-the-i-mx-8m-plus-applications-processor:8MPLUSLPD4-EVK.

[31] Jetson Nano Developer Kit. https://developer.nvidia.com/blog/jetson-nano-ai-computing/.

[32] Jetson Xavier NX Series. https://www.nvidia.com/en-us/autonomous-machines/embedded-systems/jetson-xavier-nx/.

[33] Ultra96. https://www.96boards.org/product/ultra96/.

[34] Kria KV260 Vision AI Starter Kit. https://www.xilinx.com/products/som/kria/kv260-vision-starter-kit.html.

[35] Fengou L.-C., Lianou A., Tsakanikas P., Gkana E.N., Panagou E.Z., Nychas G.-J.E. (2019). Evaluation of Fourier transform infrared spectroscopy and multispectral imaging as means of estimating the microbiological spoilage of farmed sea bream. Food Microbiol..

[36] Tsakanikas P., Fengou L.-C., Manthou E., Lianou A., Panagou E.Z., Nychas G.-J.E. (2018). A unified spectra analysis workflow for the assessment of microbial contamination of ready-to-eat green salads: Comparative study and application of non-invasive sensors. Comput. Electron. Agric..

[37] Ropodi A.I., Panagou E.Z., Nychas G.-J.E. (2016). Data mining derived from food analyses using non-invasive/non-destructive analytical techniques; determination of food authenticity, quality & safety in tandem with computer science disciplines. Trends Food Sci. Technol..

[38] He H.-J., Sun D.-W. (2015). Microbial evaluation of raw and processed food products by Visible/Infrared, Raman and Fluorescence spectroscopy. Trends Food Sci. Technol..

[39] Zhao H.-T., Feng Y.-Z., Chen W., Jia G.-F. (2019). Application of invasive weed optimization and least square support vector machine for prediction of beef adulteration with spoiled beef based on visible near-infrared (Vis-NIR) hyperspectral imaging. Meat Sci..

[40] Cheng J.-H., Sun D.-W. (2015). Rapid and non-invasive detection of fish microbial spoilage by visible and near infrared hyperspectral imaging and multivariate analysis. LWT-Food Sci. Technol..

[41] Feng C.-H., Makino Y., Oshita S., Martín J.F.G. (2018). Hyperspectral imaging and multispectral imaging as the novel techniques for detecting defects in raw and processed meat products: Current state-of-the-art research advances. Food Control.

[42] Yang D., Lu A., Ren D., Wang J. (2018). Detection of total viable count in spiced beef using hyperspectral imaging combined with wavelet transform and multiway partial least squares algorithm. J. Food Saf..

[43] Baek I., Lee H., Cho B., Mo C., Chan D.E., Kim M.S. (2021). Shortwave infrared hyperspectral imaging system coupled with multivariable method for TVB-N measurement in pork. Food Control.

[44] Guo T., Huang M., Zhu Q., Guo Y., Qin J. (2018). Hyperspectral image-based multi-feature integration for TVB-N measurement in pork. J. Food Eng..

[45] Zhuang Q., Peng Y., Yang D., Nie S., Guo Q., Wang Y., Zhao R. (2022). UV-fluorescence imaging for real-time non-destructive monitoring of pork freshness. Food Chem..

